# A Hybrid Image Filtering Method for Computer-Aided Detection of Microcalcification Clusters in Mammograms

**DOI:** 10.1155/2013/615254

**Published:** 2013-04-14

**Authors:** Xiaoyong Zhang, Noriyasu Homma, Shotaro Goto, Yosuke Kawasumi, Tadashi Ishibashi, Makoto Abe, Norihiro Sugita, Makoto Yoshizawa

**Affiliations:** ^1^Research Division on Advanced Information Technology, Cyberscience Center, Tohoku University, 6-6-05 Aoba, Aramaki, Aoba-ku, Sendai 980-8579, Japan; ^2^Graduate School of Engineering, Tohoku University, 6-6-05 Aoba, Aramaki, Aoba-ku, Sendai 980-8579, Japan; ^3^Tohoku University Graduate School of Medicine, Tohoku University, 2-1 Seiryo-mashi, Aoba-ku, Sendai 980-8575, Japan

## Abstract

The presence of microcalcification clusters (MCs) in mammogram is a major indicator of breast cancer. Detection of an MC is one of the key issues for breast cancer control. In this paper, we present a highly accurate method based on a morphological image processing and wavelet transform technique to detect the MCs in mammograms. The microcalcifications are firstly enhanced by using multistructure elements morphological processing. Then, the candidates of microcalcifications are refined by a multilevel wavelet reconstruction approach. Finally, MCs are detected based on their distributions feature. Experiments are performed on 138 clinical mammograms. The proposed method is capable of detecting 92.9% of true microcalcification clusters with an average of 0.08 false microcalcification clusters detected per image.

## 1. Introduction

Breast cancer is one of the major causes of mortality in middle-aged women, especially in developed countries [1]. At present, there are no effective ways to prevent breast cancer since its cause remains unknown [2]. Therefore, early detection becomes the key to improving the breast cancer prognosis and reducing the mortality rates. Mammography has been widely recognized as being one of the most effective imaging modalities for early detection of breast cancer. However, it is a hard work for radiologists to provide both accurate and uniform evaluation for the enormous number of mammograms generated in widespread screening. A computer-aided detection or diagnosis (CAD) system, which uses computer technologies to detect the typical signs of breast cancer, has been developed to provide a “second opinion” for radiologists and to improve the accuracy and stability of diagnosis.

**Figure 1:**
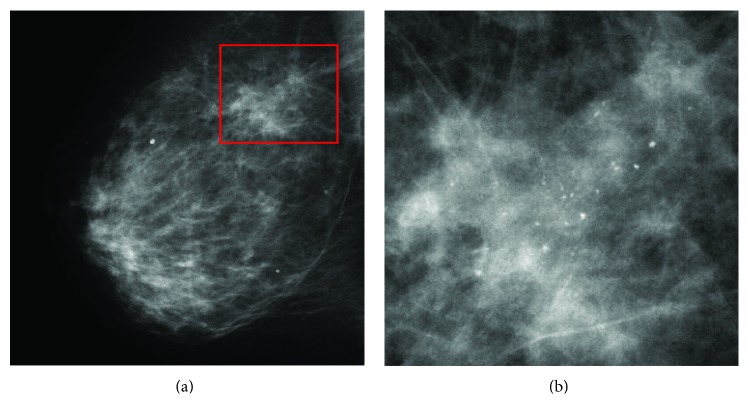
An example of an MC. (a) A mediolateral-oblique (MLO) mammogram. (b) Expanded view showing the MC.

In general, there are three signs of breast cancer in a mammogram: microcalcification clusters (MCs), architectural distortions, and masses [2]. In this paper, we particularly focus on the detection of MCs since they appear in 30–50% of mammographic diagnosed cases and show a high correlation with breast cancer [3]. According to the Breast Image Reporting and Data System (BI-RADS) lexicon [4], MCs are tiny calcium deposits that appear as small bright spots in mammograms. As an example, Figure 1 shows an MC in a mediolateral-oblique (MLO) mammogram. It is often hard for radiologists to find individual MCs in mammograms because they are very small (typically, 0.05–1 mm [3]) in the size and the contrast between the MCs and the surrounding breast tissue is not high enough. 

Over the past two decades, there has been extensive research focused on developing the CAD tools for automatic detection of MCs in mammograms. Several review papers have also been published on this topic [2, 4–8]. As described in [8], MC detection can be classified into four categories: (1) image enhancement methods [9–13]; (2) multiscale decomposition methods [14–17]; (3) stochastic modeling methods [18, 19]; and (4) machine learning methods [20–28]. In [27], a performance evaluation was summarized using free-response receiver operating characteristic (FROC) in which a support vector machine (SVM) approach [27] is found to be superior to conventional methods and is capable of achieving a true positive rate of approximately 90% when the false-positive (FP) rate is on average of 1.1 FP clusters per image. However, such FP is not low enough for clinical application. 

In this paper, we present a new method based on a hybrid method which combines a morphological technique [29] and wavelet decomposition processing for detecting the MCs in mammograms. Compared with previous methods, the proposed method can achieve not only a high sensitivity but also a lower FP. In the proposed method, we first use a multistructuring-elements (SEs-) based top-hat transform to enhance the intensity of microcalcification. Subsequently, we employ a wavelet decomposition to refine the enhanced results for removing the false enhanced microcalcifications. Then, based on the feature of malignant MCs, threshold processing is used to segment the MCs from mammograms. 

 The rest of paper is organized as follows: Section 2 presents a morphological processing technique to enhance the MCs in mammogram. In Section 3, a wavelet decomposition method is employed to refine the MCs candidates.  Section 4 presents the detection of the MCs and gives the experimental results by using the proposed method. A conclusion is given in Section 5. 

## 2. Enhancement of MCs Using Top-Hat Transform

As mentioned in Section 1, the MCs appear as small bright spots in mammograms. In this sense, MCs can be directly segmented by using a threshold process. However, since most mammograms have a low dynamical rang and the intensity contrast between MCs and surrounding tissue is quite low, selection of a threshold for the whole image is not an easy task. As a solution, the difference of Gaussian (DoG-) based method which approximates the individual microcalcification as a two-dimensional (2D) Gaussian kernel has been reported in [13], but this method only is suitable for detecting the microcalcifications with approximate circle shape. 

**Figure 2:**
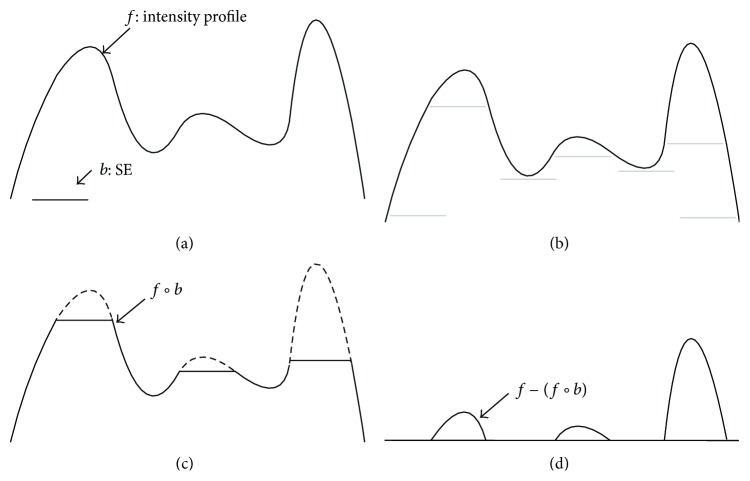
Top-hat transform in one dimension. (a) Original 1D signal and SE. (b) SE pushed up underneath the signal. (c) Opening. (d) Signal subtraction with opening result.

In this section, we propose a new method which is based on a morphological filtering technique [29] to enhance the individual microcalcifications for MC detection. The basic idea of the method is to use a set of top-hat transforms based on multi-structuring elements (SEs) of which sizes and shapes are fitted to the individual microcalcifications to enhance them. The top-hat transform of a gray-scale image *f* is defined as *f* minus its opening by structuring element *b* [30]:

(1) $$\begin{array}{c} T=f-\left(f○\;b\right), \end{array}$$

where ○ denotes the opening operation; the difference operation yields an image in which only the components fitting to the SE remain.  Figure 2 illustrates the concept of the top-hat transform in one dimension. 

**Figure 3:**
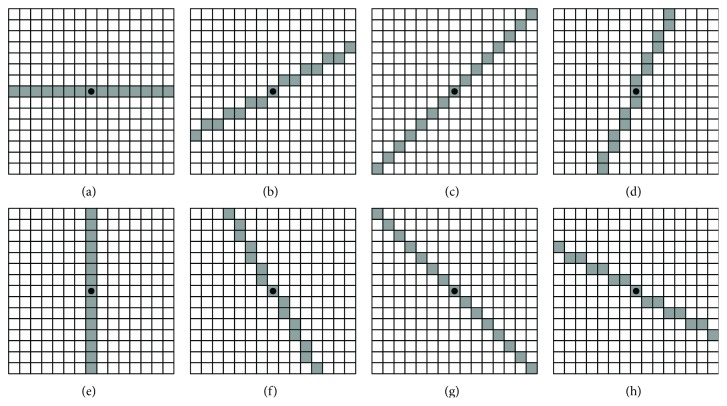
Multi-SEs converted to rectangular arrays. The dots denote the center of the SEs. These SEs are designed for fitting to the individual microcalcifications with different shapes.

In the top-hat transform, selection of an appropriate SE fitting to the target objects is the key. Since individual microcalcifications in mammograms frequently vary both in size and shape, it is imposable to use a single SE to remove all of them. To solve this problem, we use a multi-SEs-based method which uses eight different flat SEs, denoted as *b*_*i*_  (*i* = 1,2,…, 8) to remove the individual microcalcifications in the opening processing. In this paper, the SEs are a set of lines revolving around its center in a 15 × 15 array. For a mammogram image with resolution 0.05 mm/pixel, the 15 × 15 array indicates that the objects whose size are larger than 0.75 × 0.75 mm^2^ will be removed in the opening operation.  Figure 3 shows the flat SEs used in the top-hat transform where the dots denote the centers of the SEs. 

For each SE *b*_*i*_,  *i* = 1,2,…, 8, the opening operation yields an image, denoted by (*f*○ *b*_*i*_), in which the individual microcalcifications fitting to the SE are removed. Then, in order to enhance these individual microcalcifications removed by the opening operation, we use a subtraction between the original image and the maximum of the opening results to obtain an image, given by

(2) $$\begin{array}{c} E=f-\arg\max\left(f\;○\;b_{i}\right).\; \end{array}$$

**Figure 4:**
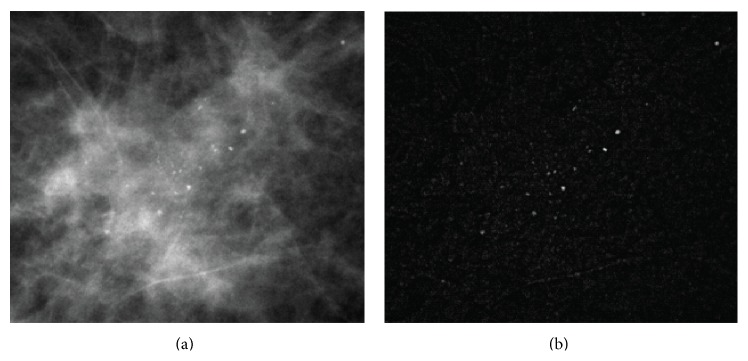
Top-hat transform enhancement of microcalcification. (a) An original mammogram image. (b) The results of the top-hat transform.

Figure 4 shows an original mammogram and an enhanced image obtained from (2). Comparing Figure 4(b) with Figure 4(a), we can see that individual microcalcifications appearing on a complex background were enhanced successfully. These results indicate that the microcalcifications can be easily segmented by using a threshold process. However, the top-hat transform also enhances some undesired objects in the mammograms, such as mammary glands, vessels, and so on. Therefore, a refinement processing is required to remove these undesirable objects from the enhanced mammogram images.

## 3. Denoising Using Wavelet Decomposition

**Figure 5:**
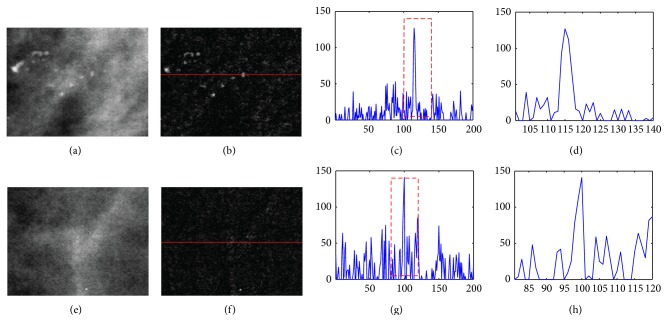
Comparison of intensity profile between microcalcification and mammary gland. (a) Microcalcifications in original mammogram image. (b) The result of the top-hat transform of (a). (c) Intensity profile of a row in (b). (d) Expanded view of the dashed rectangle in (c). (e) Mammary glands in original mammogram image. (f) The result of the top-hat transform of (e). (g) Intensity profile of the row in (f). (h) Expanded view of the dashed rectangle in (g).

As mentioned above, although the top-hat transform is capable of enhancing microcalcifications varying in size and shape, a side effect is that the undesirable objects are also enhanced. Based on an investigation, we found that these unwanted objects are mainly caused by the soft tissues, such as mammary glands and vessels [4]. The typical feature of them is that they have a relatively higher intensity compared with their surrounding area as well as an inner nonhomogeneous intensity.  Figure 5 shows a comparison between the microcalcification and the mammary gland after the top-hat transform. Comparing Figures 5(c) and 5(g), we find that the intensity of microcalcification in the enhanced image approximately equals that of the mammary glands. However, since the individual microcalcification has almost homogeneous intensity, the size of the enhanced microcalcification is generally larger than that of the mammary gland. Figures 5(d) and 5(e) show the expanded view of the dashed rectangles in Figures 5(c) and 5(g), respectively. We can find that the size of the microcalcification is almost twice as large as the size of the mammary gland. Therefore, the enhanced mammary glands can be treated as noise that can be removed according to their size. In this paper, we employ a wavelet denoising method to remove the mammary glands because the wavelet decomposition can easily separate them according to their particular size. 

The wavelet-based procedure for denoising the image consists of the following four steps.

**Step 1 .**

**Figure 6:**
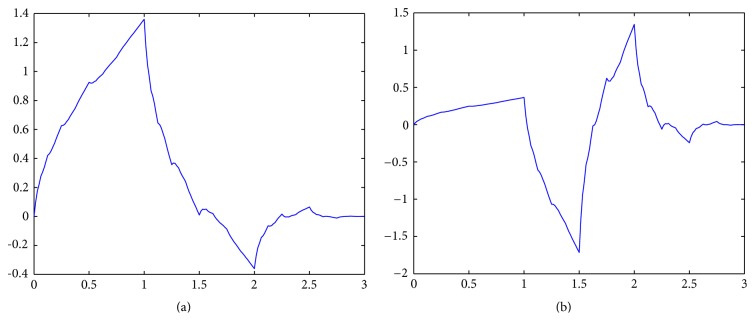
The second-order symmetrical wavelets. (a) Scaling function. (b) Wavelet function.

**Figure 7:**
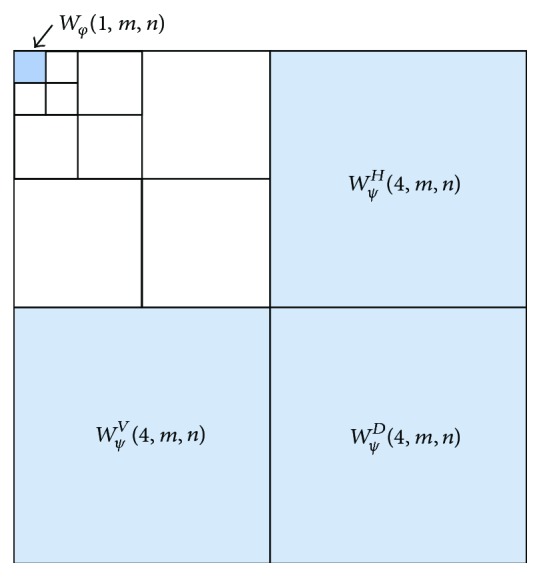
Thresholding the approximation and detail coefficients in the wavelet decomposition.

A four-scale wavelet transform is used to decompose the image obtained from the top-hat transform. We select the second-order symmetrical wavelets, short by “symlet-2,” as the decomposition filters since they have the least asymmetry and highest number of vanishing moments [31]. Figures 6(a) and 6(b) show the scaling function and wavelet function used in the decomposition filtering.  Figure 7 shows the decomposition results in which *W*_*ψ*_^*H*^(4, *m*, *n*),  *W*_*ψ*_^*V*^(4, *m*, *n*),  and *W*_*ψ*_^*D*^(4, *m*, *n*) denote the detail coefficients at level 4 size of half the original image.

**Step 2 .**

Thresholding the approximation and detail coefficients. Since the size of the microcalcification is almost twice as large as the size of the mammary gland, the noise caused by the mammary gland can be decomposed in level 4. Therefore, we set the detail coefficients at level 4 as well as the approximation coefficients at level 1 to zeros.  Figure 7 shows the result of the four-scale wavelet transform in which the shaded coefficients marked are set to zero.

**Step 3 .**

**Figure 8:**
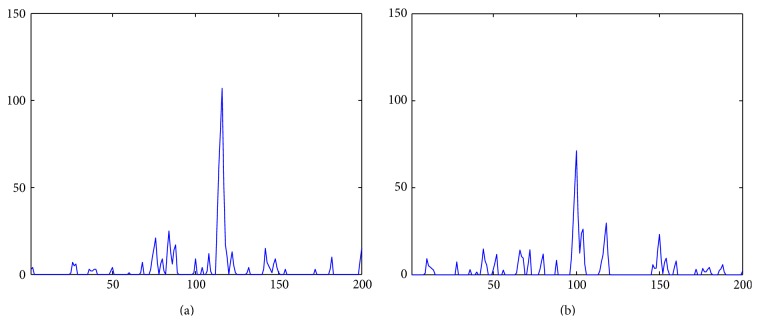
Results of the inverse wavelet transform. (a) Intensity profiles of microcalcification. (b) The intensity profiles of mammary gland candidates.

Compute the inverse wavelet transform using the modified detail coefficient. Figures 8(a) and 8(b) show the results of the inverse wavelet transform of the microcalcification and mammary gland in Figures 5(d) and 5(h). We see that the intensity of the mammary gland is clearly reduced so that the microcalcifications and mammary glands can be easily separated using a threshold.

**Step 4 .**

Segment the individual microcalcifications. In this step, we use a thresholding processing to obtain a binary image in which each microcalcification is segmented as connected components.

## 4. Detection of MCs and Experiments

In this section, we introduce a procedure that segments the MCs from the mammogram image obtained from the above sections and give some experimental results by using the proposed method. As mentioned in Section 1, the MCs are tiny calcium deposits clustering together in the mammogram images. Based on an investigation on the BI-RADS [4], we found that a typical MC generally has the following two features:

(1) an MC usually consists of four or more individual microcalcifications;
(2) an MC generally appears within a limited area of size 10 × 10 mm^2^ in mammogram images.

According to these two features, we use the following two steps to detect the MCs. First, the binary image is subdivided into blocks that overlap their neighbors both horizontally and vertically. The blocks are of size 200 × 200 pixels; for each pair of overlapping blocks, the overlapping boundary region is of size 100 × 100 pixels. Second, for each block, if the total number of the connected components is larger than three, this block will be labeled as an MC.

We developed and tested the proposed method using a database collected by the Tohoku University School of Medicine. This data set consists of 138 clinical mammograms in which 14 mammograms contain MCs. These mammograms are of size 4740 × 3540 pixels, with a spatial resolution of 0.05 mm/pixel and 16-bit grayscale. 

The performance of the proposed method is summarized by the true positive rate and false positive cluster per image.  Table 1 summarizes the experimental results of the proposed method comparing with several previous methods [8, 9, 13, 14, 32, 33]. The proposed method is capable of detecting 92.9% of true microcalcification clusters with an average of 0.08 false microcalcification clusters detected per image. To our best knowledge, this performance is better than most state-of-the-art methods in MC detection [2, 34].

**Figure 9:**
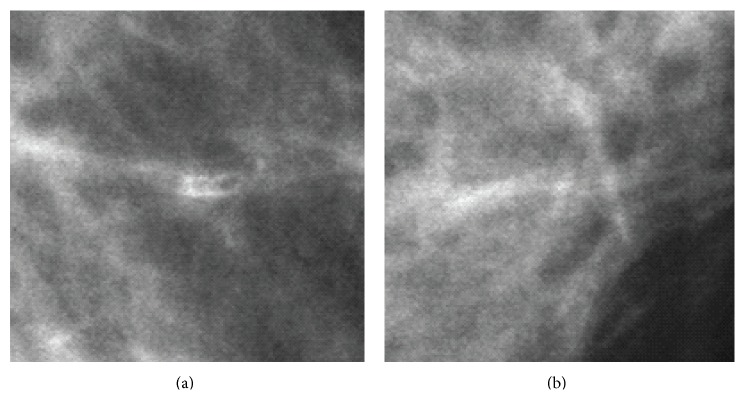
Objects causing FPs of MCs detection. (a), (b) Linear-structure tissues interlacing with each other.

In the experiments, we found that the FPs are mainly caused by the linear-structure tissues interlacing with each other or benign calcifications with nonhomogenous intensity.  Figure 9 shows three examples of these objects in mammograms. Figures 9(a) and 9(b) show the linear-structure tissues. Since the characteristics of linear-structure tissues are quite different with that of the MCs, we consider that FPs can still be reduced by using a statistic classifier, such as a support vector machine (SVM). 

## 5. Conclusion

In this paper, we presented a high-accuracy method for the detection of MCs in mammograms. The proposed method combined a multi-SEs top-hat transform and a wavelet-based denoising approach to enhance the individual microcalcifications in mammograms. Experimental results demonstrated that the proposed method is capable of detecting the MCs in mammograms with high accuracy. 

In further work, we will conduct more experiments on a wider database and improve its performance both in accuracy and computational cost.

## Figures and Tables

**Table 1:** Experimental results of MCs detection.

Methods	TP (True Positive)	FPs (False positives)/image
Difference-of-Gaussians- (DoG-) based method [13]	60%	1.2
Image-difference-technique- (IDT-) based method [9]	70%	1.5
Neural network-based method [20]	70%	1.1
Wavelet-based method [14]	75%	1.5
Support-vector-machine- (SVM-) based method [8]	90%	1.3
Weighted-local-differences-based method [32]	70%	1.9
Texture-coding-based method [33]	95%	4.3
Proposed method	92.9%	0.08
